# Associations between heavy episodic drinking and alcohol related injuries: a case control study

**DOI:** 10.1186/1471-2458-13-1076

**Published:** 2013-11-14

**Authors:** Ingeborg Rossow, Stig Tore Bogstrand, Øivind Ekeberg, Per Trygve Normann

**Affiliations:** 1 Norwegian Institute for Alcohol and Drug Research, POB 565 Sentrum, Oslo N-0105, Norway; 2 Norwegian Institute of Public Health, Division of Forensic Medicine and Drug Abuse Research, PO Box 4404, Nydalen, Oslo N-0403, Norway; 3 Emergency Department, Division of Emergencies and Critical Care, Oslo University Hospital, Ullevål, Oslo N-0407, Norway; 4 Lovisenberg University College, Lovisenberggt. 15b, Oslo 0456, Norway; 5 Department of Acute Medicine, Oslo University Hospital Ullevål, Postboks 4956 Nydalen, Oslo 0424, Norway; 6 Department of Behavioural Sciences in Medicine, Institute of Basic Medical Sciences, Faculty of Medicine, University of Oslo, Oslo, Norway

**Keywords:** Alcohol, Heavy episodic drinking, Injuries, Accidents, Violence, Case control study, Prevention paradox

## Abstract

**Background:**

Alcohol is a significant risk factor for injuries. This study addresses 1) whether the risk of alcohol related injury increases with frequency of heavy episodic drinking (HED) in a linear fashion, and 2) whether a small group of high risk drinkers accounts for the majority of alcohol related injuries.

**Methods:**

We applied a case – control design. Cases were BAC positive injured patients (n = 534) and controls were respondents to a general population survey in Norway (n = 1947). Age and gender adjusted association between self-reported past year HED frequency and alcohol related injury risk was estimated in logistic regression models for all alcohol related injuries and for violence injuries and accident injuries separately.

**Results:**

An increase in HED was associated with an increase in risk of alcohol related injury, resembling a linear risk function. The small fraction of high risk drinkers (6.6%) accounted for 41.6% of all alcohol related injuries, thus lending support to the validity of the prevention paradox.

**Conclusion:**

There is a strong relationship between frequency of heavy episodic drinking and risk of alcohol related injuries, yet the majority of alcohol related injuries are found among drinkers who are not in the high risk group.

## Background

Alcohol consumption is a significant risk factor for injuries [1], which account for a third of the overall alcohol related disease burden [2]. It is well demonstrated that the risk of injury increases with increasing amount of alcohol consumption in the hours prior to the risk situation in a dose response manner [3]. Moreover, findings from population surveys have also demonstrated that the likelihood of experiencing an injury is associated with drinking habits and increases with increasing annual alcohol intake and with frequency of heavy episodic drinking (HED) [4]. Thus, those with frequent HED are those most at risk of experiencing injuries.

However, this does not necessarily imply that prevention strategies to curb alcohol related injuries should be targeted only at the high risk group with frequent HED. If only a smaller fraction of the alcohol related injuries can be attributed to this small group of high risk individuals whereas the bulk of the harm is found among the majority of drinkers with less individual risk, it is generally argued that prevention strategies targeting the entire population of drinkers are more adequate [5,6]. If this is indeed the case, prevention efforts that mainly affect individuals at relatively low risk, may better serve the public health interest. This is referred to as the prevention paradox [5].

Whether the prevention paradox applies to alcohol related harm depends on the risk function of the association between drinking behavior and the risk of experiencing the type of harm in question. Skog [7] showed that if the risk function is - more or less - of a straight linear kind, the majority of harm can be expected to arise from the majority of low-risk drinkers simply because the majority of HED episodes are found in this group. He further argued that this type of association is most likely seen with respect to acute harms from alcohol, typically related to HED, such as accidents and violence. In line with this, many of the studies on the prevention paradox have categorized high risk drinkers in terms of frequent HED [8-12].

Most studies pertaining to assessment of the risk function of alcohol related injuries and the applicability of the prevention paradox rest, however, on general population surveys and self-reported alcohol related injuries (and other harms) [10,13,14]. While this approach to assess alcohol related harm and its association with drinking behavior is feasible and often used, it harbors significant limitations in at least two respects. First, the self-reported harms are often trivial or of little severity and may thus be of less importance to public health concern and policy initiatives. Second, the respondents’ assessments of alcohol’s role in the event of harm are, obviously, highly subjective and imprecise. It is therefore argued that assessments of the risk function of drinking behavior and alcohol related harm and the applicability of the prevention paradox should be based also on studies applying more severe injuries and objective criteria for alcohol related harms [15]. Despite the clear importance of assessment of the prevention paradox for evaluation of preventive strategies and alcohol policy making, the research literature on the prevention paradox and alcohol related harms is still fairly meager. In particular, there are so far very few studies that have addressed the applicability of the prevention paradox with respect to more severe alcohol related harms [10,15,16] and, to our knowledge, none that have addressed alcohol related injuries specifically and none that have applied objective criteria for alcohol’s involvement in the injury.

The present study aimed at addressing the validity of the prevention paradox with respect to severe alcohol related harms in two steps: 1) to assess whether the association between heavy episodic drinking and alcohol related injury resembles a linear risk function; and 2) to assess whether a larger fraction of alcohol related injuries can be attributed to a small group of high risk drinkers.

## Methods

The study applied a case–control design which is a more efficient version of a corresponding cohort study, particularly when rare outcomes are studied [17]. The study comprised two independent samples; a sample of injured patients (comprising the cases) and a sample of the general population (the controls). In both samples study participation was based in informed consent.

### Sample of injured patients

A total of 2118 adult patients (18 years or above) admitted to the emergency department of a large hospital in Oslo, Norway because of injuries from accidents, assault, or self-poisoning, were asked to participate in a study of substance use and injuries. Of these, 7% refused to participate and 4% were unable to provide blood samples (for alcohol and drug testing). Thus, the sample of injured patients comprised a total of 1882 patients (see [18] for further details). The data collection was undertaken during a 12 months period from December 2007 to December 2008. This part of the study was approved by the Regional Ethics Committee in Norway and the Norwegian Data Inspectorate. The sample comprised 58.5% men and the mean age was 51.4 years (SD = 22.6).

### General population sample

In 2012 a general population sample was contacted by Statistics Norway and asked to participate in a telephone survey on alcohol and drug related topics. The sample aimed to reflect a national representative sample of the adult population (16–79 years) in Norway and the net sample comprised 1947 respondents (response rate 53.3%). As hospital admissions due to injuries occur seldom (2 per 1000 adult inhabitants per year in Norway) and those related to alcohol occur even more seldom, we assumed that there were close to zero persons in the general population sample that had experienced a hospital admission due to alcohol related injury in the preceding year. The age group 21–30 years was over-sampled and data were thus weighted to account for design effect. The sample comprised 50.8% men and the mean age was 42.1 years (SD = 18.1).

### Measures

In both samples the participants were asked about whether they had drunk alcohol in the past year and about how often they had drunk so much that they felt clearly intoxicated in the preceding 12 months. The response categories on the latter ranged from “Zero times” to “Several times a week or more often” on an ordinal scale. Due to few observations in the upper categories, those who reported HED frequency “Several times a month”; “Once or twice a week”, or “Several times a week” were collapsed into one category of HED frequency; i.e. “Several times a month or more often” (see Table 1). The injured patients reported this in a questionnaire and they were also asked whether they had any intake of alcohol, tranquilizers and illicit drugs in the six hours prior to the injury. For each type of substance the response alternatives were: “Yes”, “No” and “Do not remember/do not know”. Patients who were not able to fill in the questionnaire were interviewed by a nurse. In some cases patient confidentiality in an interview could not be achieved in the hospital ward and for this reason self report data on frequency of heavy episodic drinking and use of substances prior to the injury are missing for 25% of the patients. Day and time of injury and type of injury were also derived from the questionnaire.

**Table 1:** Characteristics of alcohol related injured patients as compared to other injured patients (n = 1774)

	**Alcohol related injured patients n = 534**	**Other injured patients n = 1240**
Positive BAC & positive self-report, % (n)	47% (253)	n.a.
Negative BAC, and positive self-report, % (n)	31% (168)	n.a.
Positive BAC and missing (or negative) self-report, % (n)	21% (113)	n.a.
Men, % (n)	72% (386)	53% (651)
Age, mean	42 years	55 years
Poisoning, % (n)	9% (49)	5% (59)
Violence, % (n)	29% (157)	5% (59)
Accidents, % (n)	61% (328)	91% (1122)

Note: The differences in gender, age, and injury type distribution between alcohol related injured patients and other patients are statistically significant at the 0.1% level.

Blood samples were collected from the injured patients and enzymatic dehydrogenase method was used to determine blood alcohol concentration (BAC) [19], and the injury was categorized as alcohol related if the BAC level of the blood sample exceeded 0.01% or/and the patient stated that s/he had consumed alcohol within six hours prior to the injury (see [18,20] for further details). Other drugs were analysed for in whole blood using liquid and gas chromatography with mass spectroscopy. The rationale for applying self-report in addition to the objective BAC level, was that many of the patients arrived in the hospital many hours after the injury occurred, and in these cases the injury could be alcohol related but not detected in the blood alcohol determination/analysis. A previous study has demonstrated good accordance between BAC and self report of alcohol use prior to injury among patients who arrived in hospital within six hours after the injury [20].

In Norway, admission to a hospital requires referral from primary health services or ambulance services with a tentative diagnosis. Based on this diagnosis, the injuries were categorized into three broad categories; violence, self-poisonings, and accidental injuries. The violence injuries included a broad range with respect to type and severity, the self-poisonings were often overdoses of paracetamol or alcohol or other intoxicants, and the accidents included road traffic accidents, falls, and other accidents at home or in the work place.

### Statistical analyses

Only those who reported any consumption of alcohol in the preceding year or had alcohol in their blood were included in the analyses of the association between HED and risk of alcohol related injury. In the case-control design, the outcome variable is being a case or control and the cases represent being at risk of injury, whereas the controls represent not at risk. The risk curve for the association between HED and alcohol related injuries was assessed in logistic regression models, entering frequency of intoxication as categorical (with the lowest frequency category as reference group) and gender and age as co-variates. Thus the risk function was assessed as odds ratios (which correspond closely to relative risk estimates for low incident outcomes). Moreover, similar model specifications were applied to assess the association between intoxication frequency and the risk of specific types of alcohol related injury. Finally, as a previous study [15] demonstrated, some gender differences in the alcohol – injury associations such possible moderating effect was also explored in this study by including a multiplicative interaction term in the logistic regression models.

The categorization of high risk drinkers was based on the HED frequency distribution in the general population sample and a cut-off was chosen which obtained the upper 5 to 10% of the drinkers in terms of frequent heavy drinking occasions. The fraction of alcohol related injuries attributable to high risk consumers was obtained directly from the distribution of the intoxication frequency variable among the patients with alcohol related injuries.

### Sensitivity analyses

As the general population sample included respondents from all parts of Norway whereas the injured patients most probably resided in hospital’s catchment area, comprising the capital of Oslo and the surrounding county, the analyses were re-run applying data on cases and controls only from the capital area. The 2012 survey included only 380 respondents from this area, which compromised the test power for this analysis. Therefore, another data set of controls was added. This supplement data set stemmed from a general population survey in 2007 addressing alcohol and other substance use. Identical questions on frequencies of past year drinking and heavy episodic drinking were posed to these respondents and the response categories were roughly the same. This data set provided an additional 238 respondents and thus the total number of controls from the hospitals’ catchment area was 618. All analyses were re-run applying data on cases and controls from only the capital area; i.e. the city of Oslo and the surrounding county.

Another sensitivity analysis pertained to the use of other psychoactive substances than alcohol (illegal drugs and prescription drugs), as these may also increase the risk of injury [21]. As such substance use tends to co-vary with drinking behavior [22-24], this was taken into account by re-running the analyses, excluding those patients who had any illicit drug or psychoactive prescription drug in blood (see [18] for details) from the group of alcohol related injuries.

## Results

A total of 534 patients (28.4% of the sample of injured patients) were categorized as having an alcohol related injury. Among these, a majority (253 patients, 47%) had a positive BAC and reported alcohol use before the injury, another 168 patients (31%) reported such use but were BAC negative, and finally 113 (21%) had only a positive BAC (and mostly missing observations on the self-report). An alcohol related injury was more often seen among male patients (37%) than among female patients (20%) (Chi square 60.2, p < .001) and thus men constituted 72% of the alcohol related injury patients and 53% of the other injured patients. Those with alcohol related injuries were also significantly younger (mean age 42 years) than those with other injuries (mean age 55 years) (F = 144.3, p < .001). Almost one third (29%, n = 157) of the alcohol related injuries were due to violence, another 9% (n = 49) were self-poisonings, while the remaining 62% (n = 328) were due to road traffic accidents and other accidental injuries, whereas among the other injured patients the vast majority of injuries were due to accidents (91%) (Table 1).

The remaining analyses are based on cases and controls who reported to have drunk alcohol in the preceding year; 422 cases and 1725 controls (total n = 2147). The alcohol related injured patients reported significantly more frequent heavy episodic drinking than the controls (Chi square = 432.2, p < .001), (Table 2). Consequently, the relative risk (i.e. the odds ratio) of alcohol related injury was significantly higher among those who reported heavy episodic drinking several times a week as compared to those who had not had any heavy drinking episodes in the past year, after controlling for age and gender (OR = 34.4) (Table 3).

**Table 2:** Distribution of frequency of heavy episodic drinking among alcohol related injured patients and controls who reported to have drunk alcohol in the preceding year

**HED frequency**	**Among alcohol related injured patients (n = 413)**	**Among controls (n = 1692)**	**Test statistics**
None	16.5 (68)	42.7 (722)	Chi-square = 432.2, df = 5, p < .001
1-2 times per year	13.1 (54)	19.2 (325)	
3-11 times per year	15.7 (65)	26.2 (444)	
Once a month	13.1 (54)	5.3 (90)	
Several times a month	15.7 (65)	3.8 (64)	
Several times a week	25.9 (107)	2.8 (47)	

Percent (Number of observations in parentheses).

**Table 3:** Association between frequency of heavy episodic drinking and risk of alcohol related injury

	**Regr coeff**	**S.E.**	**Wald**	**OR**	**95% CI**
Intoxication frequency			293.3		
None (ref)					
1-2 times per year	0.75	0.20	13.7	2.1	1.4, 3.1
3-11 times per year	0.72	0.20	13.4	2.1	1.4, 3.0
Once a month	2.18	0.24	85.7	8.9	5.6, 14.1
Several times a month	2.84	0.25	128.9	17.1	10.5, 27.8
Several times a week	3.54	0.25	208.7	34.4	21.3, 55.6
Gender	0.31	0.14	5.4	1.4	1.05, 1.8
Age	0.024	0.004	33.0	1.02	1.02, 1.03
Constant	-4.12	0.33	159.4		

Logistic regression model; regression coefficient, standard error of estimate (SE), Wald test, odds ratio and 95% confidence interval (CI) (n = 2105).

Notes: Gender was coded 0 for women and 1 for men. Age was a continuous variable. There were no gender differences in the association between intoxication frequency and risk of alcohol related injury (the interaction term was close to zero (0.002, p = .729).

There was a clear increase in the risk of alcohol related injuries with increasing HED, and by applying a semi-continuous measure of HED frequency the risk curve fitted a straight linear form fairly well (r^2^ = 0.996). Assessment of a possible moderating effect of gender revealed no gender differences in the association between intoxication frequency and risk of alcohol related injury and the interaction term was close to zero (0.002, p = .729) (Table 3).

We further explored the associations between HED frequency and risk of alcohol related violence and risk of alcohol related accidents. These displayed similar patterns to that found for the risk of all alcohol related injuries, including straight linear risk curves (r^2^ = 0.972, and 0.965, respectively) (Tables 4 and 5). Only 37 patients with alcohol related self-poisoning had valid observations on HED frequency, and due to few observations, a similar analysis of risk of alcohol-related self-poisonings was not conducted.

**Table 4:** Association between frequency of heavy episodic drinking and risk of alcohol related violence

	**Regr coeff**	**S.E.**	**Wald**	**OR**	**95% CI**
Intoxication frequency			95.6		
None (ref)					
1-2 times per year	0.76	0.42	3.2	2.1	0.9, 4.9
3-11 times per year	0.74	0.40	3.5	2.1	1.0, 4.5
Once a month	1.91	0.42	20.2	6.7	2.9, 15.5
Several times a month	2.36	0.42	31.7	10.6	4.7, 24.2
Several times a week	3.10	0.41	57.1	22.2	9.9, 49.5
Gender	1.28	0.27	22.9	3.6	2.1, 6.1
Age	-0.04	0.01	20.2	0.96	0.95, 0.98
Constant	-4.55	0.64	51.0		

Logistic regression model; regression coefficient, standard error of estimate (SE), Wald test, odds ratio and 95% confidence interval (CI) (n = 1823).

Note: Gender was coded 0 for women and 1 for men. Age was a continuous variable.

**Table 5:** Association between frequency of heavy episodic drinking and risk of alcohol related accident

	**Regr coeff**	**S.E.**	**Wald**	**OR**	**95% CI**
Intoxication frequency			205.1		
None (ref)					
1-2 times per year	0.81	0.24	11.5	2.2	1.4, 3.6
3-11 times per year	0.76	0.24	9.8	2.1	1.3, 3.4
Once a month	2.25	0.29	59.3	9.5	5.3, 16.8
Several times a month	3.06	0.32	92.3	21.2	11.4, 39.6
Several times a week	3.78	0.30	157.4	43.7	24.2, 78.8
Gender	-0.04	0.16	0.1	1.0	0.7, 1.3
Age	0.05	0.01	97.3	1.05	1.03, 1.07
Constant	-5.50	0.41	179.8		

Logistic regression model; regression coefficient, standard error of estimate (SE), Wald test, odds ratio and 95% confidence interval (CI) (n = 1937).

Note: Gender was coded 0 for women and 1 for men. Age was a continuous variable.

Those participants who were in the upper category of the intoxication frequency variable were considered a high risk group. A total of 6.6% of the drinkers in the control group reported drinking to intoxication several times a month or more often. When applying this categorization to the group of cases, the high risk group of drinkers constituted 41.6% of all alcohol related injury patients; 48.0% of the alcohol related violence patients, and 35.5% of the alcohol related accident patients, respectively (Table 6).

**Table 6:** Distribution of alcohol related injuries by risk group and type of alcohol related injury among past year drinkers

	**Proportion of general population drinkers**	**Proportion of all alcohol related injuries**	**Proportion of alcohol related violence**	**Proportion of alcohol related accidents**
	**(n = 1692)**	**(n = 413)**	**(n = 131)**	**(n = 245)**
Low and moderate risk group; HED frequency less than several times a month	93.4%	58.4%	52%	64.5%
High risk group; HED frequency several times a month or more often	6.6%	41.6%	48%	35.5%

While gender did not moderate the association between HED and alcohol related injury, the significant gender difference in the distribution of HED frequency suggested that the proportion of alcohol related injuries attributable to high risk drinkers could be different for men and women. Among men, 9% of the population sample reported HED several times a month or more often and this high risk group accounted for 49% of the alcohol related injuries among men. Among women, almost 7% reported HED once a month or more often and this high risk group accounted for 35% of the alcohol related injuries among women (Table 7).

**Table 7:** Distribution of alcohol related injuries by risk group and gender among past year drinkers

	**Men**		**Women**	
	**Proportion of general population drinkers**	**Proportion of all alcohol related injuries**	**Proportion of general population drinkers**	**Proportion of all alcohol related injuries**
	**(n = 867)**	**(n = 293)**	**(n = 825)**	**(n = 120)**
Low and moderate risk group	90.7%	50.9%	93.1%	65.0%
High risk group	9.3%	49.1%	6.9%	35.0%

Note: For men the low and moderate risk group comprised those reporting HED frequency less often than several times a month and the high risk group comprised those who reported HED frequency several times a month or more often.

For women the low and moderate risk group comprised those reporting HED frequency less often than once a month and the high risk group comprised those who reported HED frequency once a month or more often.

### Sensitivity analyses

The analyses were re-run applying data on control subjects residing only in the capital area. Among controls, respondents living in the capital area reported on average somewhat more frequent HED occasions than others. Consequently, these analyses obtained associations between HED frequency and risk of alcohol related injuries that were somewhat attenuated, but otherwise the results were fairly similar; the associations were of a straight linear kind and less than half of the alcohol related injuries were found among the group of high risk drinkers.

A substantial proportion of the patients with alcohol related injuries had also illegal drugs and/or psychoactive prescription drugs in their blood (34%). After exclusion of these cases from the analyses, we found that the association between HED frequency and risk of alcohol related injury was of a straight linear kind, but, compared to the main analyses, the association was clearly attenuated, and a slightly less proportion of these injuries were found among the group of high risk drinkers.

## Discussion

This study showed that the risk of alcohol related injury increased as a function of the frequency of heavy episodic drinking, and we found that the prevention paradox applied; i.e. the majority of the alcohol related injury cases belonged to the large group of non-heavy drinkers, rather than to the small high risk group with frequent heavy episodic drinking.

Empirical support for the applicability of the prevention paradox with respect to alcohol related injuries is in line with what has previously been found with respect to injuries as well as other alcohol related types of harm [7-12,15]. Moreover, the study adds to a very sparse literature on the prevention paradox regarding the more severe alcohol related injuries with findings on the distribution of severe alcohol related harms in line with those of Leifman [16] and Poikolainen and co-workers [15].

### Strengths and limitations

The participation rate in the sample of injured patients was high, which is likely to reduce the risk of sampling selection bias and thus an underestimation of alcohol related injuries. Moreover, the use of blood sample analyses to assess whether the injury was alcohol related, provided an objective criterion, as compared to the subjective assessments of alcohol involvement which studies from survey data rely on [10,13,14].

On the other hand, the response rate in the general population sample was relatively low, as is generally the case in adult population surveys these days. This may well have implied that the heavier drinkers were under-represented and therefore biased the distribution of the intoxication frequency downwards. If so, the magnitude of the group of high risk drinkers will be underestimated and consequently the proportion of alcohol related injuries attributable to a small group of heavy drinkers may be overestimated.

While it may be argued that the two samples were relatively large, the breakdown into drinking behaviour categories and control for age and gender, implied fairly large confidence intervals for the point estimates and thus lessened the accuracy of the risk function, and particularly so with respect to the risk of alcohol related violence and accidents. Thus, the assessment of the risk function would have been more precise if larger samples of cases and controls had been available.

### Study implications

Empirical support for the validity of the prevention paradox implies that population strategies may be tenable to prevent or curb alcohol related injuries. Population strategies to reduce heavy drinking occasions comprise control policies to reduce demand; i.e. restrictions on physical availability, price regulations (e.g. taxation), and restrictions on advertising and promotion. These strategies are shown to be effective in reducing alcohol consumption and related harms [25,26]. Moreover, effective strategies to reduce intoxication or avoid alcohol consumption in risky situations comprise drinking driving regulations and enforcement that prevent traffic injuries [25-27] and integrated programs and enforcement in bars and pubs that prevent over-serving and violence [28,29].

Beyond the importance of effective population strategies, it may also be argued that a significant proportion may be reached by selective and indicated strategies. Regarding the former, there is good evidence that screening for harmful consumption and brief intervention in primary health care is an effective, yet not widely used measure to reduce consumption among at-risk drinkers [30,31]. Regarding indicative strategies, many patients treated for alcohol related injuries in the emergency department may also need follow up for their alcohol problems [32].

While selective and indicative strategies may not necessarily reduce injuries to any significant extent at the population level, its effect on alcohol intake and likelihood of improved health for the individual patient should also be taken into consideration [32,33]. In this context, it may be argued that the motivation to change drinking habits may be better among injured drinkers, as they have experienced adverse effects of their drinking [34].

Finally, given the importance of establishing the validity of the prevention paradox for assessment of tenable prevention strategies to curb alcohol related harm, the scarcity of empirical studies in this area calls for further research along these lines. Future studies should, amongst others, address the relationship between injury severity and the use of alcohol alone and in combinations with other psychoactive substances.

## Conclusion

There is a strong relationship between frequency of heavy episodic drinking and risk of alcohol related injuries, and those with frequent heavy episodic drinking are most at risk, yet the majority of alcohol related injuries are found among drinkers who are not in the high risk group.

## Competing interests

The authors declare that they have no competing interests.

## Authors’ contributions

IR: conceived and designed the study, participated in designing the studies from which both data sets are retrieved, performed the data analyses, and drafted the manuscript. STB: participated in designing the study from which data on cases are retrieved, participated in collecting data from cases, and participated in writing the paper. ØE: participated in designing the study from which data on cases are retrieved, and participated in writing the paper. PTN: participated in designing the study from which data on cases are retrieved, and participated in writing the paper. All authors read and approved the final manuscript.

## Pre-publication history

The pre-publication history for this paper can be accessed here:

http://www.biomedcentral.com/1471-2458/13/1076/prepub
